# A Review on the Degradation of Antibiotic Resistance Genes During Composting of Livestock Manure

**DOI:** 10.3390/toxics13080667

**Published:** 2025-08-08

**Authors:** Enwang Zhao, Yongchao Li, Jin Zhang, Bing Geng

**Affiliations:** 1 School of Environment and Natural Resources, Zhejiang University of Science and Technology, Hangzhou 310023, China; 19560127689@163.com (E.Z.); drjinzhang@aliyun.com (J.Z.); 2 Institute of Environment and Sustainable Development in Agriculture, Chinese Academy of Agricultural Sciences, Beijing 100081, China; genbing2000@126.com

**Keywords:** microbial community evolution, livestock waste, composting, antibiotic resistance genes, safety evaluation of organic fertilizers, human health

## Abstract

As emerging pollutants, antibiotic resistance genes (ARGs) have been recognized as originating from diverse sources. Among these, the use of livestock feed and veterinary drugs was identified as the primary source of ARGs in livestock manure. ARGs were found to be widely distributed in global environments, particularly in agriculture-related soils, water bodies, and the atmosphere, posing potential threats to ecological environments and human health. This paper reviewed the degradation mechanisms of ARGs during aerobic composting of livestock manure and the safety evaluation of compost products. Aerobic composting was demonstrated to be an effective method for degrading ARGs, primarily through mechanisms such as high-temperature elimination of ARG-carrying microorganisms, reduction in host bacterial abundance, and inhibition of horizontal gene transfer. Factors including the physicochemical properties of the composting substrate, the use of additives, and the presence of antibiotic and heavy metal residues were shown to influence the degradation efficiency of ARGs, with compost temperature being the core factor. The safety of organic fertilizers encompassed multiple aspects, including heavy metal content, seed germination index, and risk assessments based on ARG residues. The analysis indicated that deficiencies existed in areas such as the persistence of thermotolerant bacteria carrying ARGs, the dissemination of extracellular antibiotic resistance genes (eARGs), and virus-mediated gene transfer. Future research should focus on (1) the removal of thermotolerant bacteria harboring ARGs; (2) the decomposition of eARGs or the blocking of their transmission pathways; (3) the optimization of ultra-high temperature composting parameters; and (4) the analysis of interactions between viruses and resistant hosts. This study reviews the mechanisms, influencing factors, and safety assessment of aerobic composting for degrading ARGs in livestock manure. It not only deepens the understanding of this important environmental biotechnology process but also provides a crucial knowledge base and practical guidance for effectively controlling ARG pollution, ensuring agricultural environmental safety, and protecting public health. Additionally, it clearly outlines the key paths for future technological optimization, thus holding significant implications for the environment, agriculture, and public health.

## 1. Introduction

Antibiotic resistance genes (ARGs), recognized as emerging contaminants, induce drug resistance in microorganisms—a phenomenon of global concern [1,2,3]. Compared to antibiotics themselves, the persistence and transmissibility of ARGs in the environment pose more severe ecological threats [4]. Current research priorities focus on three core aspects: mechanisms of trans-media transmission, health exposure risks, and disruption of ecological functions. Livestock waste, a key reservoir of ARGs, contains substantial quantities of antibiotics and resistance genes, posing serious risks to ecosystems. Furthermore, ARGs can transfer to humans via the food chain, ultimately endangering human health [5,6,7,8].

### 1.1. The Use of Antibiotics in Global Animal Husbandry

Livestock feed constitutes one of the primary sources of ARGs in livestock manure. In global animal husbandry, antibiotics are commonly added to feed at low doses as growth promoters [9]. Data from the United States in 2011 revealed that the usage of veterinary antibiotics was over four times that of antibiotics used in human medicine. In 2013, the amount of antibiotics used in food-producing animals reached 14.8 million kilograms, a 17% increase compared to 2009; of this total, 80% was utilized in the agricultural sector, far exceeding the quantity used in human medicine [10]. In 2020, the average veterinary antibiotic usage in EU countries was 89 mg/kg, with tetracyclines and macrolides representing the most consumed classes. Nations such as the Netherlands and France have reduced usage through policy interventions (e.g., bans on growth promoters), though therapeutic application remains high [11]. By contrast, China’s veterinary antibiotic consumption reached nearly 30,000 t in 2018, with 53% allocated to growth promotion. China remains the world’s largest producer and consumer of antibiotics [12]. This highlights both the ubiquitous practice of using antimicrobials for growth promotion in agriculture globally and the stark contrast with bans implemented by certain economic blocs or countries. In China, over 8000 tons of antibiotics are added to livestock feed annually, with the maximum annual output of associated livestock manure reaching 3.19 billion tons [13,14]. Studies have demonstrated that the absorption rate of feed-derived antibiotics in livestock is generally less than 50%, and approximately 30–50% of these antibiotics are excreted via feces and urine in the form of parent compounds or metabolites [15,16], thereby exerting sustained selective pressure on the environment. The distribution of antibiotic resistance genes (ARGs) in Chinese livestock manure exhibits an abundance gradient characterized by poultry > swine > ruminants, with tetracycline, β-lactamase, and MLSB resistance genes being predominant. Correspondingly, antibiotic residues in manure demonstrate significant species-dependent variation, with concentrations ranking as chicken manure > pig manure > cow manure. Detection levels of typical tetracyclines and sulfonamides have reached ecological risk thresholds [13,17]. This species-specific correlation between antibiotic residues and resistance genes confirms that the persistence of antibiotic residues drives ARG enrichment, providing critical insights into antimicrobial resistance transmission mechanisms within livestock breeding environments. Annual antibiotic residues in Chinese manure total 29,000–87,000 t. Through continuous selective pressure, these residues promote ARG enrichment, as bacteria carrying resistance genes gain survival advantages and proliferate under such conditions [1,18].

Table 1 summarizes ARGs in pig, chicken, cow, and mixed poultry manure, including core types, absolute abundance ranges, key influencing factors (e.g., animal species, season, antibiotic use), treatment removal effects, and a comparison of ARG characteristics and treatment status.

Furthermore, since the 1950s, the large-scale application of veterinary drugs (including therapeutic, prophylactic, and growth-promoting uses) has significantly enhanced livestock production efficiency while concurrently releasing unmetabolized antibiotics into the environment via animal excreta [26]. This persistent pharmaceutical selection pressure drives ARG formation through a dual mechanism: (1) antibiotic residues directly induce microbial resistance mutations while simultaneously selecting for resistant strains; and (2) acting as co-selective agents, these residues amplify horizontal gene transfer of resistance determinants [27,28,29].

**Figure 1:**
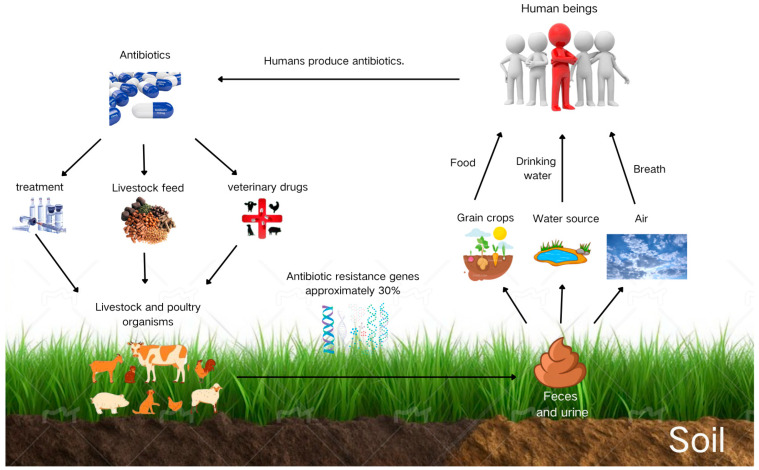
Antibiotic utilization in livestock farming, spread of resistance genes, and human exposure pathways.

Figure 1 depicts the transmission pathways of antibiotics and ARGs among humans, livestock, and the environment. Antibiotics produced by the human pharmaceutical industry are administered to livestock via feed additives, treatment, or veterinary applications, ultimately entering the animals’ systems. However, as livestock incompletely metabolize these compounds, approximately 30% of the antibiotics, along with ARGs carried by host bacteria, are excreted in feces and urine [15]. This manure enters soil systems, subsequently affecting food crops, water sources, and air quality. Humans are then exposed to ARG-containing contaminants through food consumption, drinking water, and inhalation. This exposure may lead to infections resistant to antibiotic treatment, ultimately endangering human health [6].

### 1.2. Environmental Dissemination of Antibiotic Resistance Genes in Livestock Manure

Against the backdrop of increasingly interconnected global ecosystems, the irrational use of antibiotics and veterinary drugs in rapidly developing modern animal husbandry has facilitated widespread dissemination and persistent accumulation of antibiotic resistance genes (ARGs) in key environmental compartments—soil, water, and air—posing serious threats to ecosystem stability and human health. Due to inadequate manure treatment technologies for antibiotic elimination, most antibiotics used in Chinese livestock production ultimately enter the environment [30].

Soil, water, and air serve as major ARG transmission routes. As microbial habitats, agricultural soils receive antibiotic-resistant bacteria (ARBs) from livestock feces following long-term antibiotic misuse, establishing farmland as a primary ARG reservoir [31]. ARG abundance in developed regions’ agricultural soils exceeds natural background levels by 2–4 orders of magnitude [32,33], with sulfonamide resistance genes reaching 10^−6^–10^−2^ copies per 16S rRNA in Chinese farmland soils [34]. Long-term manure application elevates ARG abundances to 30 background levels and increases detection rates of mobile genetic elements (e.g., class 1 integrons), enhancing horizontal gene transfer potential [35,36]. Regarding aquatic systems, livestock and aquaculture wastewater contain elevated ARG levels. Aquaculture-derived wastewater shows significantly higher tetracycline-related ARG concentrations than natural water bodies [37,38]. Agricultural reuse or discharge introduces ARGs into rivers, groundwater, and soils, causing secondary pollution. Antibiotics contaminate 98.0% of surface water and 96.4% of coastal seawater samples [39,40]. In atmospheric compartments, ≥16 ARG classes exist in livestock farm air, predominantly sulfonamide and tetracycline resistance genes (>200 subtypes) [41,42,43]. Ventilation systems generate ARG-carrying aerosols that disperse regionally and can be inhaled. Insufficient farm ventilation allows aerosol-bound ARGs to deposit in deep alveolar regions, disrupting respiratory and gut microbiota [44]. Enclosed farms exhibit ARG accumulation, increasing inhalation risks for workers.

ARGs demonstrate exceptional environmental persistence, particularly in soil, water, and air [32,41], necessitating targeted mitigation strategies to protect ecosystems and human health.

### 1.3. Environmental Transmission and Health Impacts of Antibiotic Resistance Genes

Bacteria carrying ARGs spread in the environment through mobile genetic elements (MGEs). Under selective pressure from antibiotics or heavy metals, these elements can integrate into the genome of recipient bacteria via mechanisms such as conjugation, transformation, or transduction, accelerating the cross-species diffusion of ARGs. The use of untreated manure or sewage sludge in agriculture introduces ARBs and MGEs into the soil. Residual antibiotics in soil can enter water bodies through leaching or runoff, exacerbating environmental selective pressure. Meanwhile, ARGs undergo horizontal transfer in water and soil via host bacteria or free DNA, ultimately threatening human health through contaminated water sources or food chains. Humans are exposed to ARGs primarily through three pathways: (1) consuming contaminated food (e.g., vegetables grown in ARG-rich soil) [40,45], with the intake of fresh vegetables cultivated in manure-amended soil recognized as a potential route for human exposure to soil ARGs [46,47]; (2) inhaling airborne antibiotic-resistant bacteria [45,48]; and (3) direct contact with contaminated water or soil [49]. The detection of ARGs homologous to those in environmental bacteria within clinical pathogens suggests that the environment may serve as a potential gene pool for clinical drug resistance, though their transmission pathways require validation through molecular epidemiological studies [50,51,52]. Murray et al. indicated via a predictive statistical model that an estimated 49.5 million deaths were associated with bacterial drug resistance in 2019, of which 12.7 million deaths were directly caused by bacterial drug resistance [53].

## 2. Degradation of ARGs During the Composting Process of Livestock Waste

Addressing ARG pollution requires efficient and feasible treatment methods. Composting technology, a waste management approach enabling resource recovery and volume reduction, utilizes elevated temperatures and microbial activity to degrade ARGs and inhibit the survival of ARG-carrying microorganisms [54]. This method provides an effective solution for reducing livestock manure-derived ARGs and mitigating the associated ecological and health hazards.

### 2.1. Composting Principle

Composting is a process where microorganisms transform organic waste into a stable, organic-rich, and environmentally friendly fertilizer [54]. This involves complex biochemical reactions and physical changes, with microorganisms playing a crucial role as the core driving force. Each microbial group possesses unique enzyme systems targeting specific organic components [55], decomposing complex constituents in organic waste (such as lignin, cellulose, and proteins) through secreted hydrolases (e.g., cellulases, kinases), and converting them into small-molecule organic compounds, carbon dioxide, water, and heat. Simultaneously, this drives the dynamic succession of pile temperature through four phases: mesophilic, thermophilic, cooling, and maturation. During this process, microbial community succession is regulated by temperature, moisture, oxygen, and carbon-to-nitrogen ratio, while adding specific microbial inoculants can accelerate decomposition, reduce ammonia volatilization, and enhance nutrient conversion efficiency. The resulting humus improves soil structure, provides plant nutrients, and enables the resource recovery cycle of organic waste. Metagenomic studies indicate that culturable microorganisms represent only 0.1% to 10% of the total microbial community [56], while metabolic complementarity of functional genes is essential for decomposing complex organics; for example, *Bacillus subtilis* secretes β-glucosidase, forming a cascade reaction with laccase produced by *actinomycetes* to break down lignocellulose into low-molecular-weight phenolic compounds, thereby providing precursors for humification [57,58]. Li et al. [59] found that livestock manure treatment removes an average of 47% of excreted antibiotics annually, with livestock farming contributing 80–88% and aquaculture accounting for 12–20% of these emissions.

**Figure 2:**
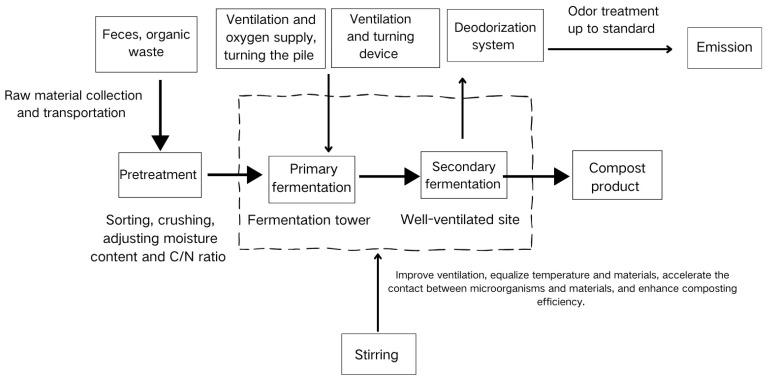
Flow chart of composting process.

Figure 2 shows the composting process flow. Firstly, raw materials were collected and transported. Manure and organic waste were pre-treated, including sorting, crushing, adjusting the moisture content, and the carbon-to-nitrogen ratio (C/N ratio). Then it entered the primary fermentation stage, which took place in a fermentation tower. During this period, ventilation for oxygen supply and turning of the pile were required. Next was the secondary fermentation, which was carried out in a well-ventilated site. Stirring operations were carried out during both fermentation stages to improve ventilation, balance the temperature and materials, accelerate the contact between microorganisms and materials, and improve composting efficiency. After that, deodorization treatment was carried out through a deodorization device. The gas was discharged after the odor met the standard, and finally, the compost product was obtained.

### 2.2. The Basic Process of ARG Removal During Composting

The composting process was typically divided into four temperature-defined stages (temperature-rises, high-temperature, temperature-decreasing, and maturity periods) that reflected microbial activity and organic matter decomposition dynamics. Temperature served as a reliable indicator of process progression because (1) distinct microbial groups exhibited specific temperature adaptation ranges; and (2) thermal variations drove successional dominance of specialized microbial communities, thereby regulating both decomposition efficiency and end-product quality. These interconnected stages collectively form the complete composting process, with each phase demonstrating unique ARG elimination patterns that are detailed in subsequent sections.

#### 2.2.1. Heating Phase

During the heating phase, mesophilic bacteria, represented by Pseudomonas and Klebsiella, dominate the hydrolysis of readily degradable carbon sources [60,61]. In the microbial community, *Firmicutes* and *Proteobacteria* have a high abundance and are the main carriers of ARGs. Among them, key genera such as *Acinetobacter*, *Sphingobacterium*, and *Lactobacillus* are significantly positively correlated with various resistance genes (e.g., tetracycline and sulfonamide resistance genes). Additionally, *Bacillus* in *Firmicutes* may carry *ermB* and *tetM* [62,63,64]. Thermodynamic analysis demonstrated that metabolic heat generation elevates pile temperature at a rate of 3–5 °C per hour [65], gradually increasing compost temperature from ambient levels to approximately 40 °C [66]. This promotes the proliferation of diverse mesophilic microorganisms, whose secreted extracellular enzymes decompose organic matter, thereby accelerating temperature rise [67]. Although this transient microbial community begins establishing competitive and symbiotic relationships [68], temperatures remain insufficient to effectively suppress ARGs. Consequently, the developing microbiota imposes relatively weak competitive exclusion against bacteria harboring ARGs [67]. Furthermore, persistent antibiotic selection pressure limits ARG attenuation due to horizontal gene transfer mediated by mobile genetic elements like integrons and transposons [69,70]. Nevertheless, ARG hosts sensitive to environmental shifts exhibit altered growth dynamics [71]. These initial metabolic adaptations establish essential preconditions for subsequent degradation processes in advanced composting stages.

#### 2.2.2. Thermophilic Phase

The thermophilic phase (45–65 °C) is the most critical stage for the reduction in ARGs during composting [72,73,74,75]. Upon entering this phase, thermophilic microorganisms become the dominant flora. Among them, *Firmicutes* still prevail (such as *Geobacillus* and *Thermus*), but their abundance starts to decrease, while *Actinobacteria* gradually increase. Key genera like *Pseudomonas* carry various ARGs, and changes in their abundance are associated with the reduction in ARGs in the thermophilic phase [67,76,77].

Thermophilic microorganisms in this phase decompose recalcitrant lignocellulosic substances using oxidases including laccase, and simultaneously secrete antimicrobial peptides to inhibit pathogen proliferation, thereby removing ARGs through multiple mechanisms: (1) directly inactivating ARG-carrying bacteria via cellular damage; (2) enhancing antibiotic degradation (with tetracycline removal rates reaching 74–92% [78]); and (3) efficiently decomposing lignocellulose (with a degradation rate > 65% [79]). The combination of sustained high temperatures and thermophilic microbial activity synergistically reduces ARG abundance through physical (thermal effect) and biological (competitive exclusion) pathways, leading to a significant decrease in ARGs (especially tetracycline resistance genes *tetM* and *tetW*) [77,80,81,82]. However, while high temperatures directly inhibit sensitive bacteria and destroy ARGs, they also cause an increased release of eARGs, which may be related to the lysis of *Firmicutes* and *Verrucomicrobia* [64]. The addition of hyperthermophilic bacterial agents can accelerate ARG removal.

It is noteworthy that high temperatures during this phase inhibit the activity of most fungi, and only a few thermophilic filamentous fungi maintain laccase activity by secreting heat shock proteins [83,84]. Metatranscriptomic data show that the expression level of cellulose-degrading genes in the thermophilic phase is 3–5 times that in the mesophilic phase [85].

#### 2.2.3. Cooling-Down Period

During the cooling phase, the temperature of compost gradually drops to ambient levels, and mesophilic microorganisms regain dominance. Among them, the abundances of *Proteobacteria* and *Bacteroidetes* rebound, with key genera such as *Arenibacter* and *Cellvibrio* co-occurring with various ARGs (e.g., *tetM*) [86]. In this phase, some ARGs (such as sulfonamide resistance genes *sul1* and *sul2*) may rebound. This is because the proliferation of bacteria after cooling leads to an increase in ARG-carrying genera (e.g., *Proteobacteria*). Meanwhile, MGEs are positively correlated with the abundance of ARGs, promoting horizontal gene transfer [63,87]. However, due to the significant reduction in ARG-carrying bacteria during the high-temperature phase, the overall abundance of ARGs remains significantly lower than the initial level [88]. It is worth noting that studies have confirmed that the inhibitory effect on ARGs persists even after the temperature drops—the ARG removal rate remains at 92% when the temperature is above 70 °C, and the durability of this inhibitory effect is stronger after ultra-high-temperature composting (90 °C) [87,89]. The stable microbial community and environmental conditions during this phase further inhibit horizontal gene transfer and the proliferation of ARG hosts, ensuring the sustained reduction of ARGs through compost maturation.

#### 2.2.4. Maturity Period

The maturation phase represents the final stabilization stage of the composting process, characterized by temperature equilibrium with the surrounding environment and reduced microbial activity. During this phase, *Actinobacteria* become the dominant phylum (e.g., genus *Streptomyces*), while the abundances of *Planctomycetes* and *Verrucomicrobia* increase. Key genera such as *Actinomadura* and *Actinotalea* are associated with the reduction in ARGs [90]. Meanwhile, actinomycetes and fungi of the phylum *Ascomycota* synergistically regulate humic acid synthesis, drive humification by degrading complex organic matter, and produce antimicrobial compounds that suppress pathogens [90,91]. Under a stabilized microbial consortium, residual recalcitrant organic matter is ultimately transformed into stable humus. The compost exhibits characteristic maturity indicators—dark coloration, friable structure, and absence of malodor—signifying humification completion.

Due to cumulative attenuation during previous phases, total ARG abundance reaches its lowest levels in this stage. However, sulfonamide resistance genes may persist, mediated by host proliferation. Nevertheless, stable physicochemical conditions coupled with microbial equilibrium further prevent ARG resurgence [92], while optimizing organic mineralization and naturally inhibiting phytopathogens [93,94]. Post-composting, the stabilized product requires safety validation prior to soil application.

### 2.3. ARG Removal Characteristics Under Different Composting Treatments

#### 2.3.1. Aerobic Composting Method

Aerobic composting represents a traditional method of decomposing organic matter through aerobic microorganism metabolism under oxygenated conditions, facilitating conversion to humus and yielding high-quality organic fertilizers [95,96,97]. While effective for ARG attenuation, removal efficiencies vary across studies. Research demonstrates 74.3% and 78.8% reductions in ARG and MGE abundances, respectively, during aerobic composting [98,99]. Ultra-thermophilic composting achieves up to 89% ARG and 88% MGE removal in sewage sludge within 21 days [87,100,101]. However, persistent ARGs post-treatment may increase under specific conditions [87,101,102,103]. For example, co-composting pig manure with food waste and sewage sludge reduced manure ARGs by 70.87%, yet increased food waste ARGs by 5.9-fold and sewage sludge ARGs by 1.3-fold [104].

Certain composting trials show significant ARG reduction after treatment, though efflux pump-related genes exhibit exceptional persistence. For instance, *ermA* became undetectable after 13–30 days, whereas *ermF* emerged as the dominant efflux-pump gene across all samples [105].

#### 2.3.2. Anaerobic Composting Method

Anaerobic composting is conducted in anaerobic or microaerobic environments, primarily achieved through sealed composting devices or controlled ventilation rates [106]. The anaerobic composting process undergoes temperature phases, including a mesophilic phase, thermophilic phase, and cooling phase, though with slower heating rates than aerobic composting. Typically, temperatures rise from ambient to 30–40 °C during the mesophilic phase and reach 40–50 °C in the thermophilic phase [107]. Studies have observed decreased abundance of various ARGs after anaerobic composting, with typical removal efficiencies of 50–60% [105,108]. However, persistence of some ARGs may occur due to bacterial host adaptability [108]. This likely stems from more complex metabolic pathways and lower energy utilization efficiency in anaerobic versus aerobic microorganisms. Under anaerobic conditions, organic matter decomposition requires synergy among multiple microorganisms, with reaction rates limited by substrate concentration and microbial population density [109,110].

Certain ARGs readily transmitted in aerobic environments may be inhibited by shifting conditions during initial anaerobic composting, causing rapid abundance decline [88]. Conversely, ARGs associated with anaerobic bacterial self-resistance mechanisms show limited removal and may temporarily increase during bacterial proliferation. For example, neither mesophilic nor thermophilic anaerobic digestion reduces *sul1* gene abundance, which becomes enriched in residual digestate [111,112].

#### 2.3.3. Natural Composting Method

Natural composting relies on naturally occurring microbial communities to passively decompose organic materials such as livestock manure and straw in open-air or simple sites. The removal efficiency of ARGs is constrained by three factors: temperature fluctuation, randomness of microbial succession, and environmental uncontrollability. Studies have shown that the removal rate of ARGs in natural composting is typically only 20–30% [92,113,114], significantly lower than that of thermophilic aerobic composting, primarily attributed to the following: (1) Lack of a sustained thermophilic phase (days with >50 °C <3 days), leading to insufficient thermal inactivation effect and high pathogen survival rates [114,115]. (2) No use of exogenous additives (such as biochar or microbial inoculants), failing to directly regulate microbial communities to inhibit ARG hosts. (3) Severe fluctuations in environmental temperature and humidity restrict the activity of key functional bacteria (e.g., cellulose-degrading bacteria) and slow the humification process.

This extensive treatment allows sulfonamide- and macrolide-type ARGs to readily undergo horizontal transfer via integrins, with their abundances rebounding by 10–20% in the late composting stage [87]. Notably, natural composting can still reduce the abundance of partial ARGs through the competitive exclusion of saprophytic bacteria, achieving preliminary control of ARG environmental risks for waste from small farms. However, applying unripe compost to rhizosphere crops should be avoided to prevent secondary soil ARG pollution [92].

#### 2.3.4. Comparison of Three Composting Methods in Degrading ARGs

Table 2 compares three composting methods, covering aspects such as advantages, disadvantages, ARG removal efficacy, and key factors influencing ARG attenuation.

Aerobic composting rapidly decomposes organic matter to produce high-quality fertilizers. Its rapid temperature elevation facilitates ARG reduction, demonstrating comparatively favorable overall performance. However, studies report variable ARG removal efficiencies, with certain resistance genes potentially persisting due to operational parameters (e.g., process specifics and additives). Anaerobic composting suits specific waste treatment, yielding functionally distinct products. Nevertheless, its slow temperature progression and organic decomposition result in inferior ARG removal relative to aerobic systems, constrained by anaerobic conditions and feedstock characteristics. Natural composting requires minimal technological complexity and achieves preliminary ARG pollution reduction. However, unregulated operation prolongs processing cycles and risks late-stage ARG dissemination. Its limited removal efficiency depends on environmental conditions and microbial succession dynamics.

Through comprehensive comparison, aerobic composting demonstrates superior ARG attenuation efficacy, establishing it as the optimal approach. Consequently, this paper specifically investigates resistance gene degradation mechanisms during aerobic composting.

## 3. Research on the Degradation Mechanism of ARGs During Aerobic Composting

Aerobic composting serves as a core technology for the harmless treatment of livestock waste, achieving the degradation of ARGs through the synergistic effects of high temperature, microbial metabolism, and physical–chemical processes [98,104]. Recent studies have shown that the removal mechanisms of antibiotic resistance genes in aerobic composting mainly involve three pathways: direct inactivation by high temperature, succession of microbial communities, and inhibition of horizontal transfer regulated by mobile genetic elements [99,117,118].

### 3.1. High Temperature Inactivates the Host and Damages DNA

High temperature serves as the primary driving force for removing ARGs during aerobic composting, where bacterial populations originally adapted to mesophilic conditions decline due to environmental changes such as rising temperatures and intensified nutrient competition, while thermotolerant bacteria gradually become dominant microbial populations [119]. These newly established dominant populations exhibit limited or no capacity to carry resistance genes, resulting in a reduction in the overall abundance of ARGs within the compost.

High temperature is the core driving force for ARG removal in aerobic composting, achieving DNA damage and inactivation of ARG-hosting bacteria through three mechanisms: (1) Thermal denaturation and oxidative damage of DNA: When the compost temperature rises to 55–70 °C, DNA double strands unwind beyond the melting temperature, shortening the half-life of ARGs from 3.9 days in conventional composting to 1.3 days [87]. Meanwhile, the production of reactive oxygen species increases by 5–7 times, causing 8-oxo-dG modification of guanine via hydroxyl radical attack and disrupting the genetic function of ARGs [62,99]. Studies have shown that ultra-thermophilic composting further reduces the half-lives of *sul1* and *tetG* to 0.9–1.1 days [87]. (2) Cell membrane disruption and intracellular ARG release: High temperature exceeds the phase transition temperature of the lipid bilayer, leading to cell membrane rupture and release of intracellular ARGs into the extracellular environment. Extracellular nucleases immediately degrade free DNA, but high temperature may simultaneously promote extracellular ARG release, with relative abundance increasing by 37% [62]. For example, lysis of Firmicutes is the main source of extracellular ARG (eARG) release, requiring prolonged thermophilic periods to reduce residual risks [62,99]. (3) Inactivation of pathogens and host bacteria: The thermophilic phase reduces the abundance of ARG-hosting bacteria such as Escherichia and Bacillus by 89%, particularly effective against bacteria carrying tetracycline- and sulfonamide-type genes [62,99]. Research shows that maintaining >70 °C for 4 days completely kills pathogens like *Escherichia coli*, cutting off the vertical transmission pathway of ARGs [99].

In aerobic composting studies of livestock manure, various ARG-carrying bacteria were detected at the initial stage, but their numbers significantly decreased after the thermophilic and maturation phases, followed by a corresponding decline in ARG abundance. Common ARG-carrying bacteria (e.g., *E. coli)* have severely limited survival capacity under high temperatures and are mostly killed. Partial studies have found that as composting progresses, the population of bacteria carrying ARGs decreases due to environmental changes such as high temperature, further indicating that high temperature plays a key role in killing ARG-carrying microorganisms and improving ARG removal efficiency. Thus, in aerobic composting, bacterial community succession triggered by temperature rise and the direct killing effect of high temperature on ARG-carrying microorganisms act synergistically, effectively reducing the overall abundance of ARGs in compost.

### 3.2. Succession of Communities Carrying Resistance Genes and Those Not Carrying Resistance Genes

Microorganisms serve as carriers of ARGs, so the succession of bacterial communities may lead to changes in environmental ARGs. Qiu et al. [117] showed that Bacteroides were significantly positively correlated with tetracycline resistance gene *tetG* and sulfonamide resistance gene *sul2*, while Corynebacterium was positively correlated with *tetG.* Xu et al. [120] found that Firmicutes showed strong correlations with ARGs during pig and chicken manure composting. The pathways of ARG degradation via microbial succession include the following: (1) Mesophilic–thermophilic succession: During the mesophilic phase, Actinobacteria and Firmicutes rapidly proliferate by metabolizing readily degradable organics like monosaccharides and starch, generating heat that raises compost temperature at 2.8–4.2 °C/h [87]. In the thermophilic phase, thermophiles become dominant, with 62–89% lower probability of carrying ARGs than mesophiles [87]. Metagenomic analysis shows that key host bacteria, such as *Gammaproteobacteria* and *Clostridia,* decrease in abundance by 89% [87]. (2) Niche competition and antimicrobial secretion: Thermophiles secrete antimicrobial peptides like thermocin to inhibit residual mesophile proliferation, blocking vertical ARG transmission. For example, in chicken manure composting, thermophilic flora reduces conjugation transfer efficiency by two orders of magnitude via 73% downregulation of type IV secretion system gene expression [62,99]. (3) Directed regulation of functional flora: Adding complex microbial inoculants (e.g., *Bacillus subtilis*) raises compost temperature to 72.1 °C, accelerating inactivation of ARG-hosting bacteria like Dietzia and Clostridium and increasing the sulfonamide gene removal rate by 24.6% [121]. One study showed that bacterial communities contributed 86% to ARG changes during municipal solid waste composting [122], while another indicated that bacterial rather than fungal communities drive ARG changes [113]. The newly formed dominant flora may carry ARGs weakly or not at all, leading to an overall decline in ARG abundance in compost.

### 3.3. Inhibiting Gene Horizontal Transfer

ARGs are primarily disseminated via vertical gene transfer (VGT) and horizontal gene transfer (HGT). HGT mediated by MGEs is the main pathway for ARG proliferation; thus, inhibiting HGT can mitigate ARG dissemination. Inhibition mechanisms include the following: (1) Thermal inactivation of integrases: Temperatures of 60 °C reduce intI1 integrase activity by 90%, significantly decreasing exogenous ARG capture efficiency [62,99]. Ultra-thermophilic composting shows a stronger correlation between MGE abundance and ARGs than conventional methods [87]. (2) Biochar adsorption blocking MGE–host contact [123], adding 2% biochar reduces MGE–host frequency by 58% through pore interception while adsorbing heavy metals (e.g., Cu, Zn) to alleviate co-selection pressure. For example, biochar-amended composting enhances *sul1* removal by 42% [101,118]. (3) Viral lysis and eARG risk control: Composting releases eARGs via viral lysis, which may be acquired by pathogens like Vibrio parahaemolyticus through natural transformation [99,124]. Membrane-covering technologies mitigate aerosol transmission and suppress MGE rebounds [125]. Wang et al. [126] observed high co-occurrence and correlation between ARGs and MGEs in pig manure composting, concluding that reducing MGE abundance lowers ARG environmental risks. Therefore, inhibiting MGEs can curtail HGT and degrade ARGs.

Based on the above three points, we can clarify that the core lies in achieving the degradation of antibiotic resistance genes through the three-level synergistic effect of high-temperature inactivation, microbial community succession, and inhibition of horizontal transfer. However, there are also some existing problems: (1) Due to the heat resistance of host bacteria, the abundance of some antibiotic resistance genes remains unchanged or even increases after aerobic composting. (2) In ultra-high-temperature composting, the removal rate of the *sulI* gene differs by 40% between 70 °C and 90 °C, and the optimal temperature and duration are still not clear. (3) The concentration of extracellular antibiotic resistance genes increases during the maturity stage of composting, and pathogens may take up these extracellular antibiotic resistance genes, thereby generating new drug-resistant strains. (4) The specific role of viruses in composting remains unclear, and further comprehensive analysis combined with metagenomics is needed.

## 4. Influencing Factors of Degradation of ARGs During Aerobic Composting

### 4.1. Key Physicochemical Parameters of Compost

Studies have reported that during aerobic composting, the degradation of antibiotic resistance genes is affected by the physicochemical properties of compost, such as temperature, carbon–nitrogen ratio, pH value, and moisture content [127,128].

Temperature was considered a key parameter and an indicator of the composting stage [88]. High-temperature composting could affect the degradation of ARGs in the following aspects: Firstly, when the temperature was at a relatively high level, the degradation ability of organic matter was enhanced. Secondly, high temperatures could reduce the number of MGEs and their host bacteria, thereby inhibiting the horizontal gene transfer of ARGs among bacteria. Thirdly, high temperature not only reduces the selective or co-selective pressure exerted on ARGs by substances such as antibiotics and hormones but also eliminates many ARG-hosting bacteria that cannot tolerate high temperatures, thus significantly reducing the abundance and content of ARGs in the compost [119]. Therefore, temperature is the core factor among the influencing factors of aerobic composting for degrading antibiotic resistance genes.

pH is an important factor affecting the growth and reproduction of microorganisms during the composting process. During the composting process, the impact of pH on ARGs is mainly determined by the growth status of host bacteria and the horizontal gene transfer of genes [129]. The optimal pH values for different host bacteria also vary. After pig manure was cultured under acidic conditions with a pH of 4.8 for 5 days, the *sul* gene and culturable sulfonamide-resistant bacteria were significantly reduced [130]. Some studies have shown that the horizontal gene transfer of ARGs was significantly promoted under acidic conditions during the composting process, while it was restricted under alkaline conditions [129]. In summary, different pH values have different impacts on different ARGs and host bacteria, but this also indicates that pH affects the degradation of ARGs during the composting process to a certain extent.

During the composting process, the moisture content directly affects the fermentation rate and compost maturity. Sha et al. [131] found that a low-moisture composting environment could inhibit the growth of potential antibiotic-resistant bacteria, and maintaining the moisture content below 30% in the final stage could prevent the spread of ARGs. If the moisture content was lower than 10–15%, the metabolic activities of bacteria would generally stop. If the water content was too high, there would be less free space in the compost pile, poor air permeability, and an anaerobic state for microbial fermentation would be formed, generating odors, slowing down the degradation rate, and prolonging the composting time.

Carbon and nitrogen are essential elements for microorganisms during the composting process. Therefore, the C/N ratio can affect the bacterial community. A study showed that an increase in the C/N ratio might increase the proportion of fungi and bacteria and enrich ARGs. An appropriate C/N ratio during the composting process (e.g., 25:1 to 35:1) is conducive to the degradation of ARGs [132]. A previous study showed that a higher C/N ratio (30:1) could prolong the high-temperature duration, thus reducing the number of pathogens carrying ARGs [133]. An excessively high or low C/N ratio may prevent the degradation of ARGs and even promote the HGT of ARGs. Thus, the C/N ratio affects the composition of the bacterial community and the community succession of ARG-hosting bacteria, thereby affecting ARGs. An excessively high C/N ratio may promote the proliferation of host bacteria, increasing the risk of ARGs spreading, while an excessively low C/N ratio may promote the horizontal gene transfer of ARGs.

### 4.2. Use of Additives

During the aerobic composting process, the application of additives exerts a significant influence on the degradation of resistance genes. These additives can be categorized into physical additives (adsorption), chemical additives (chemical reactions), and biological additives (elevating composting temperature and extending the thermophilic phase, thereby altering microbial community structure). Research indicates that additives can inhibit the production and enrichment of mobile genetic elements (MGEs), thereby controlling the HGT of antibiotic resistance genes (ARGs) while simultaneously adsorbing and degrading ARGs [134]. Furthermore, by reducing the bioavailability of heavy metals and the effective carbon content, additives can further enhance the removal efficiency of ARGs during composting, leading to a reduction in their abundance [135,136]. For instance, Qian et al. [137] found that the addition of biochar, fly ash, and zeolite significantly reduced the abundance of six types of ARGs and two types of MGEs. Lu et al. [138] reported that adding ferric chloride to a pig manure composting system not only increased methane production but also reduced ARGs by 33.3%.

Concurrently, additives can alter the distribution of bacterial communities, potentially indirectly influencing the antibiotic-related ecological environment by modulating bacterial species composition and abundance. They can suppress the production and enrichment of MGEs, thereby controlling the HGT of ARGs; reduce the concentrations of antibiotics and heavy metals, alleviating selective pressure on resistance genes and fundamentally weakening the drivers for their existence and dissemination; and directly adsorb and degrade ARGs to reduce their abundance [134].

Specifically, biochar achieves enhancement through temperature regulation, microbial modulation, and chemical adsorption [118]. Thermophilic inoculants can increase the peak composting temperature by 8.1 °C, shorten the composting period to 9 days, and improve the removal rate of β-lactam antibiotic resistance genes by 23.8% [121]. Although red mud aids in nitrogen retention, it reduces the removal efficiency of antibiotic resistance genes by 34% and thus requires cautious application [139]. Novel additives like nanoscale zero-valent iron remain in the experimental stage, enhancing reactive oxygen species generation through Fenton reactions [125]. By altering environmental factors, additives can affect the behavior and fate of ARGs in the environment. Integrating these mechanisms, additives play a crucial role in eliminating antibiotics, offering multiple potential pathways and directions for addressing issues related to antibiotics and associated resistance genes.

Table 3 lists the degradation rates of different additives for the degradation of antibiotic resistance genes during composting. It can be seen from the table that the ultra-high-temperature microbial community has the best improvement effect. Therefore, further research can be conducted on the process parameters and conditions of ultra-high-temperature composting to achieve the optimal removal effect of resistance genes.

### 4.3. Heavy Metal Residue

Since the last century, studies have confirmed the co-evolutionary relationship between heavy metals and ARGs. Partial research indicates that ARGs are positively correlated with heavy metal concentrations [13]. Heavy metal pollution is often accompanied by high abundances of ARGs, a correlation rooted in heavy metals forming co-selection pressure through co-regulation of efflux pump genes and physical linkage of resistance genes [113]. For example, Zhu et al. studied large-scale pig farms in China and found that ARG abundances in manure were enriched by up to 28,000-fold compared to control samples, showing a significant positive correlation with Cu and Zn concentrations. Meanwhile, transposase gene abundances increased by 90,000-fold, indicating that heavy metals indirectly promote ARG diffusion by activating HGT [1]. This finding was further validated by Liu et al. in pig manure composting: each 1 mg/kg increase in bioavailability of exchangeable Cu and Zn led to a 15–22% increase in intI1 abundance, thereby accelerating the spread of ARGs such as *tetM* and *sul2* via conjugal transfer [147].

During agricultural waste (AFW) composting, the presence of heavy metals significantly influences ARG abundances [61], exerting a dual effect on ARG dynamics: (1) Direct selective pressure: Heavy metals (e.g., As^3+^) induce oxidative stress to activate the SOS repair system, increasing bacterial genome mutation rates by 3- to 5-fold and directly driving the emergence of novel ARGs [147]. (2) Indirect ecological interference: Guo et al. showed via metagenomic analysis that Cu pollution increased the abundance of multidrug resistance genes in compost while significantly raising the proportion of potential pathogenic hosts [148]. This process relates to heavy metals inhibiting the activity of organic matter-degrading bacteria (e.g., *Bacillus*), causing microbial communities to shift toward resistant species [64]. Heavy metals interfere with the removal efficiency of ARGs during aerobic composting through the above mechanisms.

## 5. Environmental Risk Assessment of ARGs in Composted Organic Fertilizer

Compost has gained widespread use as a soil amendment due to its demonstrated agronomic benefits, including high nutrient content and reduced pathogenic bacteria [149,150,151,152]. However, studies have shown that applying immature or unstable compost products can negatively impact plant growth through phytotoxic compounds or suboptimal growth conditions [153,154]. These findings underscore the importance of conducting thorough safety evaluations before agricultural application to safeguard both soil ecosystems and crop quality.

### 5.1. Conventional Safety Evaluation of Organic Fertilizers

The conventional safety assessment of composted organic fertilizers follows China’s Organic Fertilizer Standard NY 525-2021 [155]. This evaluation examines fundamental physicochemical properties, including pH, organic matter content, and total nutrient levels (N-P-K). Particular attention was given to heavy metal contamination, with concentrations of regulated metals such as Cd, Pb, As, Hg, and Cr being carefully monitored due to their environmental persistence and potential ecological risks [156,157,158]. The germination index (GI) served as a primary indicator of compost maturity and biotoxicity, where values exceeding 60% were generally considered acceptable for agricultural use [159].

da Luz T M F N assessed the health risks associated with reusing manure as agricultural fertilizer via an Environmental Risk Assessment, finding that zinc was the metal contributing most significantly to the risk of organic waste, rendering the risks posed by toxic cadmium and lead almost negligible [160]. Li et al. evaluated compost products by the Organic Fertilizer Standard NY 525-2021 stipulated by the Ministry of Agriculture and Rural Affairs of the People’s Republic of China [161]. They observed that the contents of zinc and copper in the fermented products exceeded the limits specified in the standard, while the nickel content met the requirements. Additionally, the number of fecal coliforms in the fermented products was found to be less than 100, and the mortality rate of Ascaris eggs exceeded 95%, both of which complied with the standard limits. In Li et al.’s study, the germination index (GI) values of compost products were 92.7% and 90.5% [161], respectively, which were higher than 60%, indicating that the fermented products had good maturity and were non-toxic to rapeseed. In Oktiawan et al.’s experiment using mung bean plants, the GI increased by 74.9%, which was higher than 60%, suggesting that the phytotoxicity of the compost had dissipated and the compost was mature [162]. At this stage, compost products can be applied to soil as organic fertilizers.

### 5.2. Risk Assessment Based on ARG Residue

Current research has established frameworks for assessing ARG-related risks in compost products. When ARGs persist in organic fertilizers due to incomplete composting, their subsequent application may facilitate dissemination in soil ecosystems through HGT. This process increases the risk of resistance acquisition among soil microbial communities, potentially compromising crop safety and ecosystem stability [163]. Metagenomic evidence indicates that while composting reduces ARG abundance compared to raw manure, residual levels remain significantly higher than background soil concentrations [164]. These studies particularly highlight HGT between non-pathogens and pathogens as the primary driver of resistance evolution in agricultural environments.

The global assessment of ARG risks focuses on three critical dimensions: (1) human accessibility (potential for environment-to-human transmission), (2) mobility and pathogenicity (transfer to pathogenic hosts), and (3) clinical relevance (association with medically important antibiotics). Recent investigations have further established that global patterns of antibiotic usage directly correlate with ARG-related health risks [165]. These findings underscore the need for advanced monitoring approaches, such as the integration of metagenomics with machine learning, to better predict and manage the dissemination of resistance.

## 6. Current Challenges and Future Research Directions

Aerobic composting faces challenges in managing ARGs. Thermotolerant bacteria preserve or increase some ARGs, while high temperatures release eARGs that risk secondary contamination. Key needs include optimizing UHTC parameters (e.g., for *sulI* and *vanA*), developing eARG inactivators, and exploring viral–ARG interactions via metagenomics and viromics.

(1) Due to the thermotolerance of host bacteria, the abundance of certain ARGs remains unchanged or even increases after aerobic composting, leading to low removal efficiency of persistent ARGs. Ultra-high-temperature composting (UHTC) can decompose thermotolerant bacteria; optimal additives can be identified through experimental testing for the targeted removal of these bacteria.
(2) High temperatures during aerobic composting release eARGs, whose concentrations increase during the maturation phase. Pathogens may uptake eARGs, generating new drug-resistant strains and causing secondary contamination. It is necessary to identify or develop eARG inactivators to specifically block eARG dissemination; validate the DNA cleavage efficiency of nanoscale zero-valent iron to reduce eARG abundance; and investigate bacteriophage lysins for targeted clearance of drug-resistant hosts.
(3) The optimal temperature–duration combination for UHTC remains unclear: sulfonamide resistance gene (*sulI*) removal rates differ by 40% between 70 °C and 90 °C, yet the ideal temperature–duration parameters are undefined. A temperature–duration coupling model should be established to determine optimal degradation conditions for *sulI* and vancomycin resistance gene (*vanA*), such as maintaining 80 °C for 120 h.
(4) Bacteriophages carry tetracycline resistance genes; however, research on viral communities in composting remains limited. Critical mechanisms underlying the roles of viruses in composting constitute a knowledge gap, necessitating comprehensive analyses that integrate metagenomics and viromics. Viromics should be employed to track associations between bacteriophages and ARGs in compost, thereby elucidating the interaction mechanisms between viruses and ARGs.

## 7. Conclusions

The presence of ARGs in livestock waste poses a serious threat to the ecological environment and human health. ARGs originate from a wide range of sources and are distributed in soil, water, and atmospheric environments. Comparative studies have shown that aerobic composting exhibits a better effect on degrading resistance genes, primarily through mechanisms such as bacterial community succession, high-temperature inactivation of ARG-carrying microorganisms, reduction in the abundance of host bacteria, and inhibition of horizontal gene transfer. However, factors including the physicochemical properties of aerobic composting systems, the use of additives, and residues of antibiotics and heavy metals can affect the degradation efficiency of ARGs. Meanwhile, safety assessments of compost-based organic fertilizers (encompassing indicators such as heavy metal content and seed germination index) and risk assessments based on ARG residues indicate that products derived from aerobic composting can be applied to soil as organic fertilizers.

## Figures and Tables

**Table 1:** Comparison of ARGs in different animal manures.

Animal Type	Core ARG Types	Absolute Abundance	Key Influencing Factors	Removal Effect of Treatment Technologies on ARGs	References
Pig manure	1. Tetracyclines (*tetM, tetW*) 2. Macrolides (*ermB, ermF*) 3. Sulfonamides (*sul1, sul2*)	10^5^–10^8^ copies/g	Abundance in summer is higher than in winter Antibiotic use significantly increases multidrug resistance genes	Composting: Overall abundance reduced by 12–96%, but *ermC* may proliferate	[19,20,21]
Chicken manure (broiler/layer)	1. Sulfonamides (*sul1*) 2. βlactams (*blaCTXM*) 3. Multidrug resistance genes (*mefA, acrB*)	2.8 × 10^5^–7.8 × 10^5^ copies/g	ARG diversity in broiler manure > layer manure Directly driven by feed additives (e.g., chlortetracycline)	Composting: Sulfonamide ARGs reduced, but vancomycin gene (*vanA*) abundance increased	[22,23]
Cow manure (dairy cow/beef cattle)	1. Tetracyclines (*tetQ, tetO*) 2. Aminoglycosides (*aadA, strB*) 3. Chloramphenicols (*cmlA*)	2.1 × 10^5^–3.3 × 10^5^ copies/g	β-lactamase genes are still detected in cow manure without antibiotic use ARG abundance in dairy cow manure < beef cattle manure	Composting: Tetracycline gene abundance reduced to 10^−10^ copies/16S rRNA	[21,22]
Mixed poultry manure	1. Multidrug resistance genes 2. Quinolones (*qnrS*)	ARG concentration in PM2.5: 3.94 × 10^3^ copies/m^3^ Potential pathogens in manure: 44 types	Aerosolization of manure leads to airborne transmission of ARGs *Escherichia coli* and Shigella as the main hosts	Constructed wetland: Removal rate of *sul1* and *tetA* 12.3–39.2% Plant absorption (e.g., reed): Overall removal rate > 60%	[23,24,25]

**Table 2:** Characteristics of different composting treatments on the removal of antibiotic resistance genes.

Composting Method	Characteristics							Degradation Rate of Resistance Genes	Key Factors Affecting ARG Removal	References
	Oxygen Supply Requirement	Treatment Cycle	Temperature Range	Dominant Microbial Community	Pathogen-Killing Effect	N/C Loss	Disadvantages			
AerobicComposting	Requires forced ventilation or turning for oxygen supply	3–6 months	50–70 °C	*Firmicutes*, *Actinomycetes*, *Thiobacillus*	High temperature can effectively kill pathogens	High nitrogen loss (20–40%), high carbon loss (40–60%)	High energy consumption may produce odors	70–90%	Physicochemical properties, additive application, antibiotic/heavy metal residues	[97,98,100,102,104,105,106]
Anaerobic Composting	No need for oxygen supply, requires a sealed environment	6 months to several years	<40 °C	*Methanobacterium*, *Clostridium*	Almost unable to kill pathogens	Low nitrogen/carbon loss	Slow decomposition may produce methane and odors	50–60%	Microbial community, temperature change, product accumulation, and composting raw material characteristics	[92,107,108,109,110,111]
Natural Composting	No need for oxygen supply, no manual intervention	1–2 years	No stable high temperature	Opportunistic pathogens, resistance gene hosts, and parasite eggs	Unable to kill pathogens	Loss degree is uncertain, but degradation efficiency is low	Low degradation efficiency, easy to breed pests and pathogens	20–30%	Natural environmental conditions; Natural succession of the microbial community	[92,113,114,116]

**Table 3:** Degradation rates of resistance genes by different additives.

Additive Type	Specific Substance	Target ARGs	Removal Rate	Core Mechanism	Reference
Biological Additives	Hyperthermophiles	Total ARGs	89%	High temperature destroys DNA structure and inhibits host reproduction	[87]
	Compound Microbial Inoculants	*sul1*, *sul2*, *tetA*	90%	Increases *Bacillus* abundance and accelerates nitrogen fixation	[63]
Physical Additives	Biochar (Rice husk-derived)	*sul1*, *intI1*	84.3%	Adsorbs extracellular DNA, reduces HGT	[101]
	Biochar (Mushroom residue-derived)	Total ARGs	78%	Dose and feedstock-dependent	[140]
	Bamboo Charcoal	Total ARGs	54%	High specific surface area adsorbs	[141]
	Natural Zeolite	Total ARGs	19%	Limited effectiveness	[142]
	Struvite-loaded Zeolite	*intI1*, *sul1*, *tetG*	60%	Reduces the bioavailability of contaminants	[143]
Chemical Additives	Nano Zero-Valent Iron (nZVI)	*tet(W)*	86.6%	Generates reactive oxygen species to destroy DNA	[144]
	Micro-scale Zero-Valent Iron	Tetracycline/glycopeptide	90%	Inhibits host metabolism; effectiveness > nano-scale iron	[143]
	Magnetic Fe_2_O_3_/Red Mud Nanoparticles	*ermB*	85%	Iron ions interfere with electron transfer	[145]
	Lime Nitrogen (CaCN_2_)	Total ARGs	63.5%	Releases cyanamide to inactivate gut microorganisms	[146]

## Data Availability

No new data were created.
